# Non-Solvent Influence of Hydrophobic Polymeric Layer Deposition on PVDF Hollow Fiber Membrane for CO_2_ Gas Absorption

**DOI:** 10.3390/membranes12010041

**Published:** 2021-12-28

**Authors:** Abdul Latif Ahmad, Amir Ikmal Hassan, Leo Choe Peng

**Affiliations:** School of Chemical Engineering, Engineering Campus, Universiti Sains Malaysia, Nibong Tebal 14300, Malaysia; amirikmal88@yahoo.com (A.I.H.); chcpleo@usm.my (L.C.P.)

**Keywords:** poly(vinylidene fluoride) membrane, polypropylene, hydrophobic coating, non-solvent

## Abstract

The implementation of hydrophobicity on membranes is becoming crucial in current membrane technological development, especially in membrane gas absorption (MGA). In order to prevent membrane wetting, a polypropylene (PP) dense layer coating was deposited on a commercial poly(vinylidene fluoride) (PVDF) hollow fiber membrane as a method of enhancing surface hydrophobicity. The weight concentration of PP pellets was varied from 10 mg mL^−1^ to 40 mg mL^−1^ and dissolved in xylene. A two-step dip coating was implemented where the PVDF membrane was immersed in a non-solvent followed by a polymer coating solution. The effects of the modified membrane with the non-solvent methyl ethyl ketone (MEK) and without the non–solvent was investigated over all weight concentrations of the coating solution. The SEM investigation found that the modified membrane surface transfiguration formed microspherulites that intensified as PP concentration increased with and without MEK. To understand the coating formation further, the solvent–non-solvent compatibility with the polymer was also discussed in this study. The membrane characterizations on the porosity, the contact angle, and the FTIR spectra were also conducted in determining the polymer coating properties. Hydrophobic membrane was achieved up to 119.85° contact angle and peak porosity of 87.62% using MEK as the non-solvent 40 mg mL^−1^ PP concentration. The objective of the current manuscript was to test the hydrophobicity and wetting degree of the coating layer. Hence, physical absorption via the membrane contactor using CO_2_ as the feed gas was carried out. The maximum CO_2_ flux of 3.33 × 10^−4^ mol m^−2^ s^−1^ was achieved by 25 mg modified membrane at a fixed absorbent flow rate of 100 mL min^−1^ while 40 mg modified membrane showed better overall flux stability.

## 1. Introduction

The fabrications of highly hydrophobic membranes are not uncommon in current membrane technology advancement. Liquid-to-gas contact applications such as membrane gas absorption (MGA) or membrane distillation (MD) utilize this type of membrane to maintain high performance at long periods of operations [1,2,3]. This is because in longer operations, membranes used tend to get extremely wetted due to long exposure to absorbents used [4,5]. Membrane wetting is a common cause for the disruption of separation performance due to the intrusion of liquid absorbents into the membrane’s pores [6]. This phenomenon can be prevented by focusing on improving the membrane’s hydrophobicity. Hydrophobicity is the determination of membrane resistance towards wetting and represents its effectiveness through water contact angle values [7]. Hence, the implementation of high hydrophobicity or superhydrophobic (contact angle: >150°) on membranes to attain low surface free energy has become a requirement [8].

The use of low surface energy membranes which exhibit hydrophobic properties owing to high intrinsic water angles such as poly(vinylidene fluoride) (PVDF), polypropylene (PP), and polytetrafluoroethylene (PTFE) also helps in building wetting resistance [9,10,11,12]. Khaisri et al. [7] compared all three commercial membranes, and PTFE has the highest water contact angle of 133.5° as compared to PP and PVDF, of which the water contact angles are averaged at 104° and 92°, respectively. In a study by Ahmad et al. [13], PVDF possesses lower contact angles averaged at 80°–100°. Therefore, in this case, PTFE is considered to have the highest wetting resistance. However, as studied by Khaisri et al., commercial PVDF and PP membranes are proven to be much more cost-efficient and also retain low surface energy, which is sufficient for liquid–gas contact applications. Accordingly, PVDF is known to have stable chemical and mechanical properties, which are suitable for various applications [14]. Thus, the selection between PP and PVDF as a base substrate is often debated. Even so, the utilization of a single-base substrate is essentially not adequate. Therefore, several modifications such as functionalization, surface modification, and additive enhancement were conducted in previous works as an attempt to reach the optimum hydrophobic state [15,16,17].

Surface modification is a method that is usually employed on commercial membranes to improve its surface characteristics. This is usually achieved by implementing grafting, plasma treatment, or surface coating. Erbil et al. [18] utilized the addition of varied non-solvents separately, such as methyl ethyl ketone (MEK), cyclohexanone, and isopropyl alcohol (IPA) into a coating solution of dissolved granular PP. A homogenous gel-like coating solution was formed and deposited on a glass slide. A water contact angle of 160° was achieved, rendering the surface formed superhydrophobic. This concept was then followed by other researchers such as Lv et al. [19], which adapted the formulation and deposited a coating solution on PP hollow fiber membranes. The base substrate with a contact angle of 122° was enhanced to a contact angle of 156°, forming a superhydrophobic membrane (contact angle: >150°) [8]. Atomic Force Microscopy (AFM) parameters were also implemented in the study to evaluate the membrane’s surface roughness. The results showed an alteration of the modified membrane surface roughness, exhibiting 12.2 times higher Root Mean Square (R_ms_) value as compared to pristine membrane conditions.

This leads to another method derived, where non-solvents are separated from mixing with a coating solution [20]. A flaw was identified, where premature precipitation due to the fact that the reaction between the polymer coating solution and the non-solvent occurs during the coating process [21]. Therefore, a sequential dip-coating procedure is implemented, where the membrane is first dipped into the polymer coating solution followed by the non-solvent. Himma et al. [22] employed this two-step sequential dip-coating procedure on a PP membrane as a strategy to obtain coating homogeneity in their study. The nature of this method allows more flexible control of coating methods, primarily dip-coating [17], which then progresses into more unconventional coating such as vacuum-coating [15]. Conventionally, dip coating is favorable to form thinner coating layers which minimize the mass transfer resistance [17]. This benefits applications that involve the transportation of gases such as MGA due to the low resistance across the membrane.

Recent studies covered the potential PP coating efficiency on PP membranes by using the selection of non-solvents such as ethanol, IPA, acetone, MEK, and cyclohexanone exclusively [22]. Among the selection used, MEK induces the formation of smaller homogenous microspherulites on the modified membrane’s surface, which produces the best contact angle of 149°. However, there are literature gaps that were not explored regarding PVDF membranes on the non-solvent compatibility with the PP polymer coating. The combination of two distinct low-surface-energy polymers forming composite membranes is possible to increase the membrane’s surface hydrophobicity by adapting methods from previous works. Therefore, in this study, the potential usage of a commercial hollow fiber PVDF membrane with a PP polymer coating was investigated. Two-step dip-coating was used to deposit a PVDF hollow fiber membrane into MEK as the selected non-solvent, followed by a PP coating solution. The weight concentration of PP granules in forming the coating solution was varied. The membranes with and without the non-solvent were also investigated. The non-solvent interaction with the coating solution was investigated to understand the formation of the hydrophobic surface. The membrane characterization such as the contact angles, FTIR spectra, and SEM images of the surface and the cross-section were also conducted. To finalize both pristine and the modified hollow fiber membrane performance evaluation, MGA involving distilled water as a liquid absorbent was utilized to determine the membrane coating wettability characteristics. This study aimed to investigate the PP coating solution compatibility with commercial PVDF hollow fiber membranes using MEK as a non-solvent. The coating capability of the modified membranes was then tested to withstand wetting through physical absorption for CO_2_ capture.

## 2. Materials and Methods

### 2.1. Materials

A commercial-grade PVDF hollow fiber membrane (MSFUF1040) was used as a substrate for the deposition of a hydrophobic coating, which was provided by IT Tech Research, Malaysia. Xylene (analysis grade; Merck, Darmstadt, Germany) was used as the polymer solvent to dissolve commercial PP granules (Sigma-Aldrich, Darmstadt, Germany). MEK (>99.5%; Merck) was used as the non-solvent.

### 2.2. Preparation of the Hydrophobic Surface

The preparation of a hydrophobic coating solution using PP polymer granules was adapted from the formulation by Himma et al. [17]. The granule concentrations varied from 10 mg mL^−1^ to 40 mg mL^−1^ were added to a flask and dissolved slowly in xylene solution. The flask was then placed in a heating mantle filled with a glycerin bath. The solution was then heated to 110 °C, while it was constantly stirred with a magnetic stirrer until it dissolved completely and a homogenous colorless solution formed. Prior to dipping, the ends of the PVDF hollow fiber membranes were sealed with epoxy resins to prevent the coating solution from entering the membrane. Two-step immersions were then implemented, where the PVDF hollow fiber membrane anchored at a steel rod was first dipped in the non-solvent solution for 30 s followed by the dissolved PP solution for 10 s. The immersion step was conducted by hand entirely. The sample was then dried under vacuum at constant temperature around 40–50 °C for 3 h.

### 2.3. Characterization

#### 2.3.1. SEM

The membrane sample’s surface was characterized using a scanning electron microscope (SEM, Hitachi TM 3000). All samples were thin-coated with gold/palladium using a sputter coater (Quorum SC7620) for 90 s prior to analysis.

#### 2.3.2. Contact Angle Measurement

The contact angles of deionized water droplets on the surface of the PVDF hollow fiber membrane were analyzed using a contact goniometer (Rame-Hart 250-F1, Succasunna, NJ, USA). The membrane was placed onto the edge of a glass slide with a double-sided tape. Two microliters of deionized water droplets were dropped using a microsyringe at room temperature. The contact angles were then calculated via a digital video image using graphics software program, DropMeter A-100. To minimize errors and deviating results, average readings were taken on 5 different spots on the same hollow fiber surface. Each reading was taken 30 s after the droplets were dropped on the membrane.

#### 2.3.3. Bulk Porosity

The bulk porosity, *ε_b_*, of the substrate layer was determined via dry–wet weights measurement. The samples were immersed in distilled water for 24 h. The weight of the wet samples was taken after excess water on the outer surface was wiped off using a filter paper. The wet samples were then dried in an oven for 24 h, before their weights were then recorded. The equation used to calculate the porosity as follows [23]:

(1) $$\varepsilon_{b}=\frac{\frac{W_{w}-W_{d}}{\rho_{w}}}{\frac{W_{w}-W_{d}}{\rho_{w}}+\frac{W_{d}}{\rho_{p}}}\times 100\%,$$

where *ε_b_* is the bulk porosity, *W_w_* is the wet membrane weight (g), whilst *W_d_* is the dry membrane weight (g), ρ_*w*_ is the density of water (1.00 g cm^−3^), and ρ*_p_* is the polymer density used (1.78 g cm^−3^).

#### 2.3.4. FTIR

An FTIR spectrometer (Thermo Scientific Nicolet Nexus 670, Waltham, MA, USA) was utilized to examine the presence of the selected functional groups on the membrane surface. The samples were scanned over the wavenumber range of 650 cm^−1^ to 4000 cm^−1^. Average spectra of 32 scans were recorded per membrane sample.

#### 2.3.5. Experimental Setup of the MGA Process

In order to conduct the performance comparison between the membranes, pristine, and modified PVDF hollow fiber membranes, these membranes were assembled in a hollow fiber module separately. The module ends were sealed with epoxy resins to prevent any gas or liquid leakage during experiment. The detailed specifications of the commercial PVDF hollow fiber membrane are specified in Table 1 below. The specifications of the membrane module for MGA is presented in Table 2 below.

**Figure 1:**
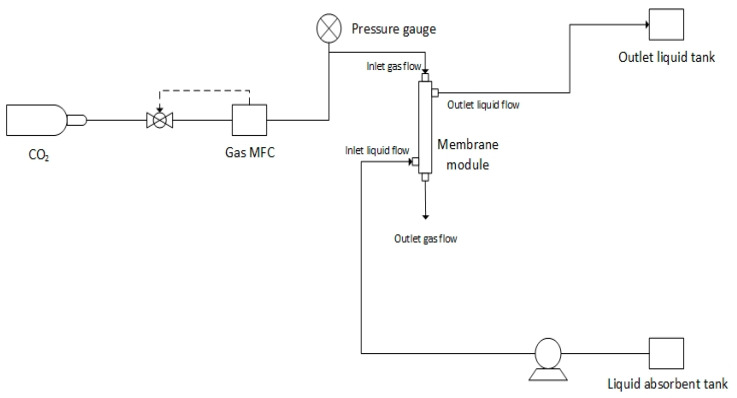
Schematic drawing of the MGA setup.

Figure 1 shows the schematic diagram of MGA for the CO_2_ gas absorption process. The module configuration was set up to flow in a countercurrent flow. The gas phase entered from the lumen side of the membranes, and it was released into the environment from the bottom module outlet. The liquid phase flowed countercurrent through the shell side of the membrane. The gas used in the experiment was pure CO_2_ gas, and the gas flow was controlled with a mass flow controller (MFC, AALBORG 0–500 mL min^−1^) depicted in figure below. The liquid absorbent used in this case was distilled water, and it was pumped into the shell side of the membrane using a peristaltic pump from a liquid absorbent tank. The outlet liquid absorbent was then collected in an outlet liquid tank. The feed gas flow rate and the liquid absorbent flow rate were fixed at 120 mL min^−1^ and 100 mL min^−1^, respectively. The gas side pressure was controlled to be at 1 × 10^5^ Pa, and the liquid phase pressure was controlled to be 0.2 × 10^5^ Pa higher than the gas phase in order to prevent any bubble formation in the liquid phase [24]. In every run, the system was allowed to stabilize for 30 min before taking any experimental data. The results of each run were averaged from three times of sampling; each sampling was taken every 30 min. All experiments were carried out at an atmospheric pressure (101 kPa) and at room temperature (25 °C).

#### 2.3.6. Mass Transfer Rate of CO_2_

The separation performances for each pristine and the modified PVDF hollow fiber membranes were evaluated using the following equation below [25]:

(2) $$J_{CO_{2}}=\frac{Q_{l_{in}}\times C\;}{A},$$

where $J_{CO_{2}}$ is the CO_2_ flux (mol m^−2^ s^−1^); $Q_{l_{in}}$ is the inlet liquid flow rate (m^3^ s^−1^); *C* represents the CO_2_ concentration (mol m^−3^), and *A* represents the effective area of the membrane in the module (m^2^). The concentration of CO_2_ in water was determined with the titration method, where NaOH (0.001 M) was titrated into the collected distilled water sample of 15 mL every 30 min, and it was determined as follows [26]:

(3) $$C=\frac{MW_{g}\times NaOH\;molarity\times Volume\;of\;NaOH\;titrated\;\left(L\right)}{Volume\;of\;distilled\;water\;sample\;\left(L\right)},$$

and the effective area of the membrane was calculated as [27]:

(4) $$A=n\pi d_{o}L,$$

where *n* is the number of fibers used for the experiment, $d_{o}$ is the outer diameter of the membrane, and *L* represents the effective membrane length used.

## 3. Results and Discussion

### 3.1. PP Coating Solution Interaction with and without the Non-Solvent PVDF Hollow Fiber Membrane Surface Morphology

A preliminary study was first conducted in the absence of the non-solvent on the PVDF membrane to witness the changes on the membrane’s surface. The PVDF samples were straight dipped into the coating solution, skipping the sequential non-solvent dipping step. The PP coating solution was kept at 75–80 °C so as to maintain the solution liquidity and preventing gelation [17]. In addition, a low-base substrate contact angle also amplified the wetting of the coating solution on the membrane’s surface [28]. This eased PP deposition and allowed the solution to homogenously spread across the membrane for the precipitation of a coating layer.

**Figure 2:**
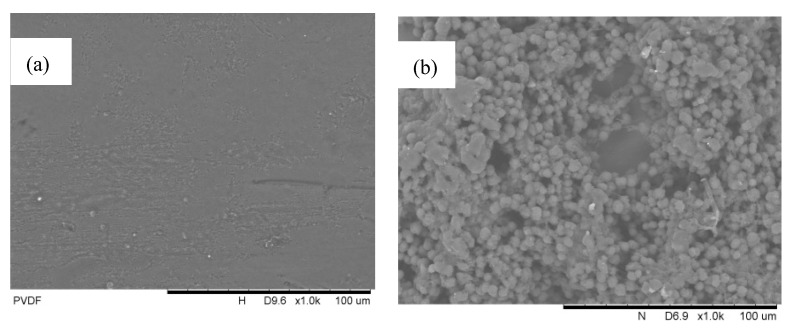
SEM surface morphology images of the pristine membrane (**a**) and 10 mg mL^−1^-polypropylene (PP)-concentration PVDF hollow fiber membrane PVDF (**b**) at a 1000× magnification without the non-solvent.

The SEM surface morphologies of a 10 mg mL^−1^-PP-concentration PVDF hollow fiber membrane samples without dipping in the non-solvent are shown in Figure 2 below. Figure 2a depicts a smooth PVDF membrane surface prior to modification. There was no coating layer present on the surface of the membrane inducing any extra thickness. Contrary to the result in Figure 2b, the aggregations of microspherulites were observed to form non-uniformly. This could be explained that the pre-coating of the membrane in the absence of the non-solvent induced the uncontrollable crystallization of microspherulites. The aggregations of PP spherulites were concentrated on a spot, stacking on one another. This caused pores of the membranes to be blocked, which limited the full potential of the coating and the membrane to be used in an application that requires high membrane porosity. Similarly, this was observed at higher PP concentrations, but with more aggressive spherulites aggregation.

Thorough coating evaluation was then conducted in the presence of the non-solvent, which was MEK in the coating procedure, by employing the two-step immersion method—dipping initially in the non-solvent followed by dipping in the coating solution. The temperature of MEK was kept at room temperature (25 °C), while the temperature of the PP coating solution was maintained at 75–80 °C. This was to prevent any instance of the rapid dissolution of the PVDF membrane into the PP solution, causing a disoriented coating layer [29]. Theoretically, MEK is not a non-solvent for PVDF, since it acts as a solvent to a certain degree [30]. Evidently, this is also proven by Bottino et al. [31], where the solubility of PVDF was tested on 64 different solvents. Based on that study, MEK is classified as a good swelling agent by Hansen solubility parameters (HSPs), which holds as neither a good solvent nor a good non-solvent. Considering the interaction between the solvent, non-solvent, and polymer substrate is being discussed, the HSP values of the three components are crucial for chemical suitability. These values are used to predetermine the polymer solubility with the solvent used. *δ_t_* was determined with the equation below:

(5) $$\delta^{2}{}_{T,p}=\delta^{2}{}_{D,p}+\delta^{2}{}_{P,p}+\delta^{2}{}_{H,p}.$$

The values are obtained from the radius of the sphere in the Hansen space referring to coordinates that corresponds to the polymer. Upon the identification of both the polymer and solvent coordinates, Hansen parameters in the Hansen space (*R_a_*) were calculated as follows [32]:

(6) $$R_{a}=\sqrt{\;4\times {\left(\delta_{Ds}-\delta_{Dp}\right)}^{2}+{\left(\delta_{Ps}-\delta_{Pp}\right)}^{2}+{\left(\delta_{Hs}-\delta_{Hp}\right)}^{2}}.$$

A soluble solvent upholds *R_a_* larger than the interaction radius (*R_o_*) value; hence, a relative energy difference (RED) number was relatively used to quantify the distance from *R_a_* to *R_o_* [33]:

(7) $$RED=\frac{R_{a}}{R_{o}}.$$

A RED number of 0 signifies no energy difference; a RED number of <1 is an indication of high polymer-solvent affinity, while a RED number of >1 indicates lower affinity and a RED number of ~1 is a borderline between the lower and higher affinities. Table 3 below summarizes the HSP values of PVDF, MEK, and xylene. Taking account of HSP values represented in Table 1, the RED number for each component was also determined in Table 3 below.

From the RED values obtained in the table, PVDF did not dissolve in both xylene and MEK at any circumstances due to having higher RED values than 1. However, MEK upheld a RED number at a borderline between having properties of a solvent and a non-solvent. Therefore, the precautionary action of the temperature and time of exposure to MEK and the coating solution was controlled to prevent any reaction of the polymer substrate prior to coating layer formation.

**Figure 3:**
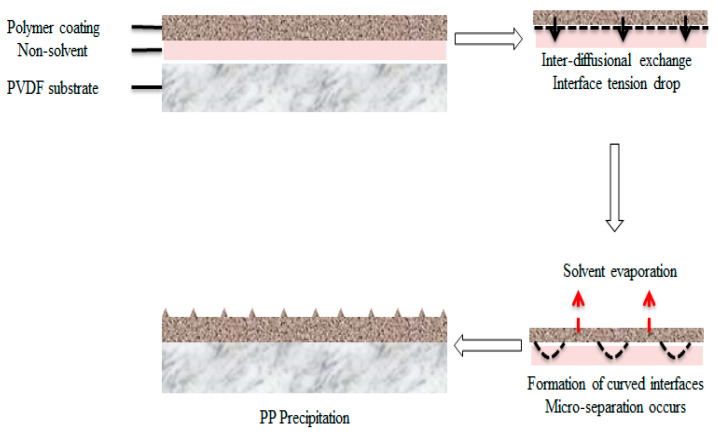
Precipitation of a polypropylene (PP) coating from the non-solvent–solvent interaction on the PVDF hollow fiber surface.

MEK in this case acted as a precipitator to induce coating layer crystallization and homogeneity [35]. This explained the scarcity of PP microspherulites, when the membrane was coated in the absence of MEK. When the PP solution was coated on the PVDF membrane’s surface where the non-solvent is present, a solvent–non-solvent reaction occurred. An interface between the two was formed, and inter-diffusion took place. Interface tension gradually dropped, leading to the formation of curved interfaces. In this case, it underwent phase separation, when the solvent exchanged into the non-solvent. The presence of the non-solvent produced two macroscopic phases, i.e., rich polymer phase and poor polymer phase. The concentrated polymer phase establish a continuous matrix, whereas the poor polymer phase formed pores where eventually PP precipitates, forming distribution of coarse PP coating layer [36]. This interaction is portrayed in Figure 3 below. In addition, high MEK volatility also played an important role in the precipitation surface structure. A high evaporation rate led to a faster solidification step, giving insufficient time of proper formation, resulting in smaller spherulite aggregates and a smoother surface [17]. Notably, MEK is known to give the best homogeneity as compared to other non-solvents used in previous studies, which is evenly and densely distributed microaggregates [19,22,37].

MEK has shown changes on the membrane surface morphology owing to high hydrophobicity characteristics in different studies conducted by other researchers [17,18]. However, that is in the case of a PP coating solution on PP membranes. Essentially, the hydrophobic polymer coating is influenced by the solubility parameters between the coating solution, the non-solvent, and the polymer substrate [22]. Therefore, in this study, the compatibility of MEK with the PVDF membrane and the PP coating solution played a vital role in the crystallization of the coating layer on the membrane’s surface.

**Figure 4:**
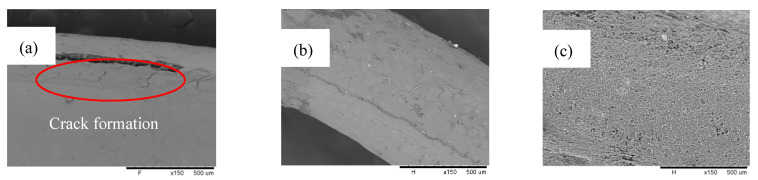
SEM surface morphology images for the modified membranes with the non-solvent at different concentrations: (**a**) 10 mg mL^−1^; (**b**) 25 mg mL^−1^; and (**c**) 40 mg mL^−1^. A magnification of 150× was used for each concentration.

Upon the formation of the PP layer on the membrane’s surface, the SEM surface morphology images of the modified membrane with different concentrations of MEK starting from 10 mg mL^−1^ to 40 mg mL^−1^ are as shown in Figure 4 (from left to right). From the figures, distinctive features were shown between the PVDF membranes dip coating with the non-solvent and without the non-solvent. A solid layer was present for all polymer coating weight concentrations, confirming the presence of a PP layer on the surface. However, there seemed to be a crack formation for 10 mg mL^−1^, which could be the result of the instability of MEK with PP during the phase separation, leading to an uneven coating of the surface [38]. Tomar et al. suggest that this phenomenon is possible to happen during the drying of the polymer substrate after it is coated [39]. MEK evaporation leads to film shrinkage that enables the formation of a solid-like film, which induces residual stress within the film. Upon different overviews, Zuri et al. explained the possibilities of the solubility parameter between the solvent and the polymer substrate, which plays a role in inducement of stress [40]. Better chemical compatibility results in a better adhesion of the polymer coating on the polymer substrate. In this case, the occurrence of coating cracks was uncommon and not seen in other PP concentrations. Hence, it could be a buildup of residual stress within the coating layer due to heating, instead of chemical incompatibility.

On the other hand, microspherulites were not apparent on the surface of the membrane at low PP concentrations, contrary to the study made by Franco et al. [37]. PP microspherulites started to form at a 25 mg mL^−1^ PP concentration, revealing patches of a small spherulites surface on the membrane, while also covered with a smooth solid layer. As the PP concentration became as high as 40 mg mL^−1^, a highly aggressive spherulites formation was shown (Figure 4c). A dilute-polymer-concentration solution formed a thinner and porous skin layer, as compared to a higher-concentration solution. Furthermore, a higher PP concentration formed a viscous solution, which slowed down the diffusional exchange rate of PP with MEK, yielding to slower precipitation. This led to denser and much aggressive polymer aggregates, which can be an offset to porosity for the benefits of a thicker coating layer [41].

**Figure 5:**
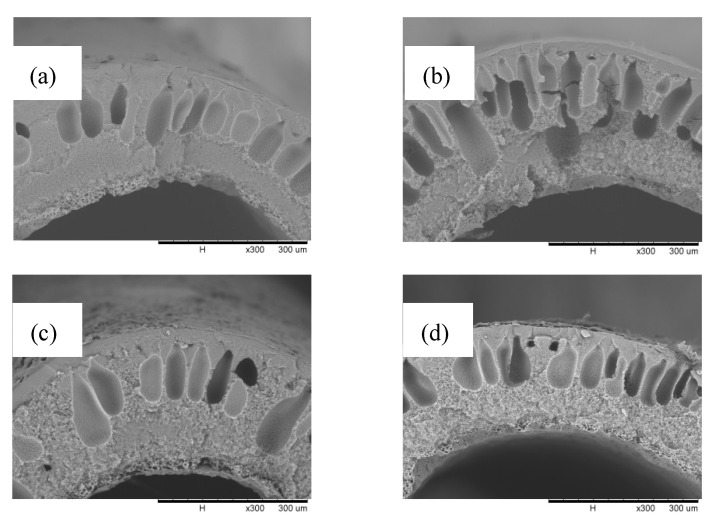
SEM cross-section images for the pristine membrane (**a**) and the modified membranes with MEK at concentrations of 10 mg mL^−1^ (**b**), 25 mg mL^−1^ (**c**), and 40 mg mL^−1^ (**d**). A magnification of 300× was used for all concentrations.

A closer inspection was performed in Figure 5, representing the cross-sections of all modified membranes at all concentrations and the pristine membrane. The formation of a coating layer was more clearly observed on all modified membranes as compared to that of the pristine membrane, exhibiting a neat surface. At MEK concentration of 10 mg mL^−1^, a coating layer was present with a thickness of 2.4 µm. This was followed by the coating layer with a thickness of 3.95 µm at a MEK concentration of 25 mg mL^−1^ and the coating layer with a thickness of 4.04 µm at a MEK concentration of 40 mg mL^−1^. The thickness of the coating layer was taken by averaging those at three different spots, and as expected, a coating layer at a MEK concentration of 40 mg mL^−1^ was slightly thicker, considering it was more concentrated. However, distinct features were apparent even from the cross-sectional view where the membrane’s surface started form small spherulites as the PP concentration increased. Definitive spherulite formation at a MEK concentration of 25 mg mL^−1^ can be clearly seen from the side view of the cross-section, proving MEK influence on the coating layer precipitation.

### 3.2. Characterization of the Modified PVDF Hollow Fiber Membrane with and without the Non-Solvent

#### 3.2.1. Bulk Porosity and Contact Angle

**Figure 6:**
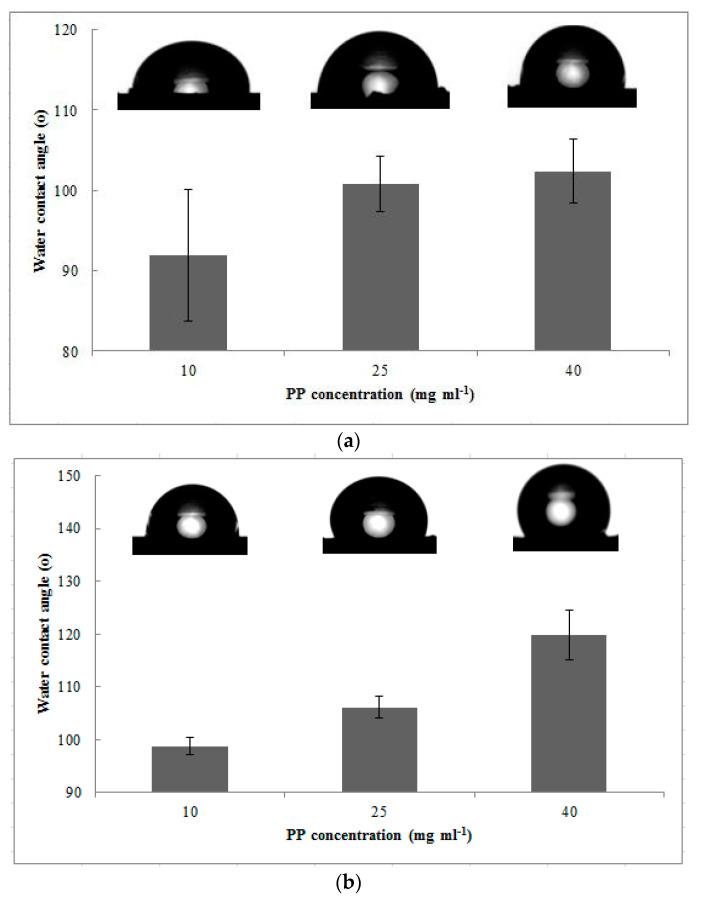
Contact angles of the modified PVDF hollow fiber membrane without MEK (**a**) and with MEK (**b**). The corresponding images of water droplets are depicted for each concentration.

To assess the effective pores and hydrophobicity of the modified PVDF hollow fiber with and without MEK, a summary of porosity and contact angles was shown in Table 4 below. Hydrophobicity is often measured using the value of the contact angle. According to Kadir et al. [42], a contact angle higher than 90° is a clear indication of the achieved state of hydrophobicity. Hence, the contact angle for each concentration is depicted in a bar graph, along with the corresponding water droplet on the membrane surface without MEK in Figure 6a and with MEK in Figure 6b, to determine its hydrophobicity. Initially, the pristine PVDF hollow fiber membrane held a basis of a porosity of 89.42% and a contact angle of 72.53°. Comparing that of the base membrane to the porosity values displayed in the table below, the modified membranes without MEK were very low in porosity as compared to the membranes with MEK. Although the stacking of PP spherulites were concentrated only on certain areas on the modified membranes without a MEK surface, there were possibilities where the coating solution seeped into an uncovered area of the membrane pores [15]. Consequently, this led to the fact the blockage of pores stemmed from aggressive aggregations of PP spherulites, as portrayed in Figure 2b. Hence, in the presence of MEK as the non-solvent, the membrane was maintained at high porosity. At PP concentration of 40 mg mL^−1^, the bulk porosity peaked at 87.62%, and at PP concentration of 25 mg mL^−1^, which is stated to be the optimum PP concentration, a 80.27% bulk porosity was reached [37]. Theoretically, MEK’s nature was dispersive, when it interacted with its immiscible polymer, in this case, PVDF at ambient conditions [43]. Subsequently, MEK thoroughly coated the hollow membrane curved surface, mitigating the PP coating solution seeping into the pores. Considering higher porosity at 40 mg mL^−1^ PP concentration, it could be detrimental for liquid–liquid or gas–liquid applications such as MGA and MD [44]. The inability to prevent liquid intrusion into the membrane pores reduced the absorption flux of gas penetrants into the system over a long period of time.

Generally, rougher surface attributes to higher hydrophobicity [45]. The contact angles of the modified and unmodified membranes were heavily linked with a jagged surface. The pristine PVDF hollow fiber membrane averaged at a 72.53° contact angle, and as the concentration increased, the contact angle gradually increased [46]. Objectively, at a PP concentration of 40 mg mL^−1^, the highest contact angle reached up to 119.85°, signifying high hydrophobicity as compared to a lower PP concentrations. This corresponded to its membrane surface structure in Figure 4c, completely covered by the PP coating surface. On the contrary, the membrane surface structure at a PP concentrations of 25 mg mL^−1^ did not exhibit the same surface structure where the spherulites formation was dominant, hence granting fewer hydrophobicity characteristics. In comparison with previous studies, the coating of PP was deposited on the PP hollow fiber membranes, which initially possessed high hydrophobicity, enabling simpler methods to achieve high hydrophobicity [8]. However, in this case, reaching high hydrophobicity for the current pristine PVDF hollow fiber membrane with a relatively low contact angle demands an effective surface modification.

By correlating the results of the SEM images, contact angle measurements, and porosity, the fibers modified with the non-solvent favorably showed better hydrophobicity and the coating layer homogeneity. The modified membranes without MEK rendered an ineffective coating due to inconsistent PP microaggregations on the PVDF membrane surface. Hence, the presence of the non-solvent as the precipitator helped to develop stability for PP solidification and obtain surface homogeneity, although the compatibility between PVDF and MEK was still arguably undignified with the selected parameters.

#### 3.2.2. Characterization of the FTIR Spectra of the Modified PVDF Hollow Fiber Membrane with and without the Non-Solvent

**Figure 7:**
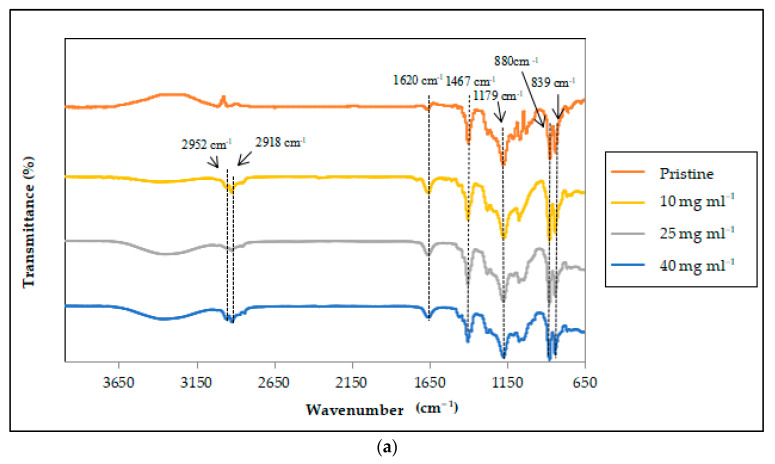

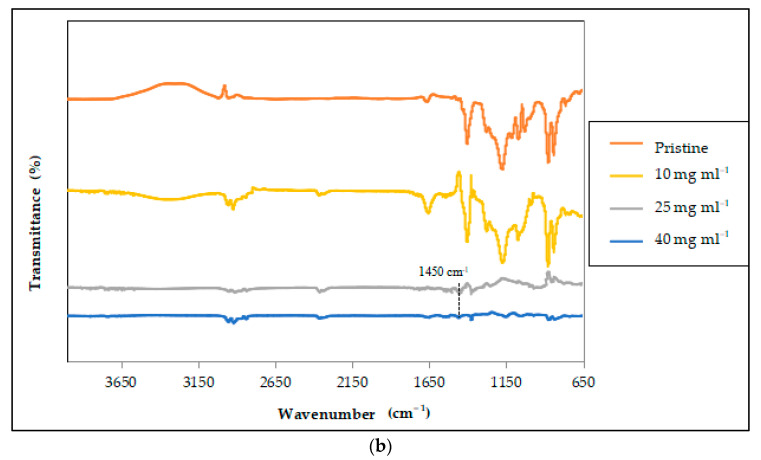
FTIR spectra of the modified PDVF hollow fiber membrane without MEK (**a**) and with MEK (**b**) as the non-solvent for all PP weight concentrations.

The modified PVDF hollow fiber membranes were characterized with FTIR to verify the presence of a PP coating layer by assessing the characteristic bands that were generated. Figure 7a,b depicts the characteristic bands of the modified membranes without MEK and with MEK, respectively. Comparing all the FTIR spectra in Figure 7a, the pristine PVDF membrane may be attributed to the C=C stretch at 1620 cm^−1^, which was visible for all weight concentrations. There were strong absorption bands at 1467 cm^−1^, 1179 cm^−1^, 880 cm^−1^, and 839 cm^−1^ for the pristine PVDF membrane. These bands were also present and identical with the proceeding modified membrane PP concentrations. The difference between the spectra was the intensity of the peaks. However, newly resembled peaks at 2952 cm^−1^ and 2833 cm^−1^ of the C–H bond that were not present in the pristine PVDF spectrum may be due to the presence of the PP coating formation that overlapped with PVDF bands. The stretch from 2950 cm^−1^ to 3250 cm^−1^ is a common indication of the C–H stretch, which is prominent for PVDF membranes [47]. Its distinctive peaks represented by its intensity, shape, and position is the basis of FTIR spectroscopy. Nevertheless, the bands for PP absorption were identical to those investigated in previous studies [48,49]. This confirmed the success of a PP coating layer, even though it is not dominant in absorption bands due to the inhomogeneous deposition on the membranes surface. The distinct intensity difference in the absorption bands of homogenous PP deposition can be observed in Figure 7b.

Contrary to the spectra in Figure 7a, non-identical absorption bands for the modified membranes with MEK at 25 mg mL^−1^ and 40 mg mL^−1^ PP weight concentrations were detected when compared with the pristine PVDF spectrum in Figure 7b. The peaks at 2952 cm^−1^ and 2833 cm^−1^ were also identified in this state, which further confirmed the PP coating presence. Even so, PP characteristic peaked at 1450 cm^−1^ with the vibration of CH_3_ and –CH_2_ around the 1470–1370 cm^−1^ region. The flat distorted spectra from 1370 cm^−1^ to 650 cm^−1^ conformed to the PP resemblance by previous work [49]. However, modified membranes PP concentration of 10 mg mL^−1^ with MEK still possessed the PVDF substrate characteristics with a slightly different intensity due to the formation of PP coating. Nonetheless, the resemblances of PP peaks were identical for 25 mg mL^−1^ and 40 mg mL^−1^ PP weight concentrations with MEK, and the previous peaks produced for the pristine PVDF membrane were no longer detected. Hence, this could be a clear sign of a complete covering of the PP layer on the membrane’s surface, reducing the intensity of the peak-resembling indication of PVDF. Hence, the peaks can be strengthened with proper coating consistency by enabling surface homogeneity for more accurate representation of data.

### 3.3. CO_2_ Absorption Performance of the Pristine and Modified PVDF Hollow Fiber Membranes

To evaluate the hydrophobicity and coating layer performances of the membranes, physical CO_2_ absorption with distilled water was conducted using MGA at room temperature (25 °C). The operation was conducted in a period of 1 h 30 min, subjected to three times liquid absorbent sampling. All membrane samples were conducted in similar conditions. The modified hollow fiber membranes without MEK were not taken into account for MGA evaluation due to its coating instability and credibility, as mentioned in Section 3.1. This included modified membranes with MEK at 10 mg mL^−1^. It had the lowest contact angle as compared to those with MEK at 25 mg mL^−1^ and 40 mg mL^−1^. Hence, the pristine and modified membranes with MEK at 25 mg mL^−1^ and 40 mg mL^−1^ were chosen and evaluated for MGA experiments.

**Figure 8:**
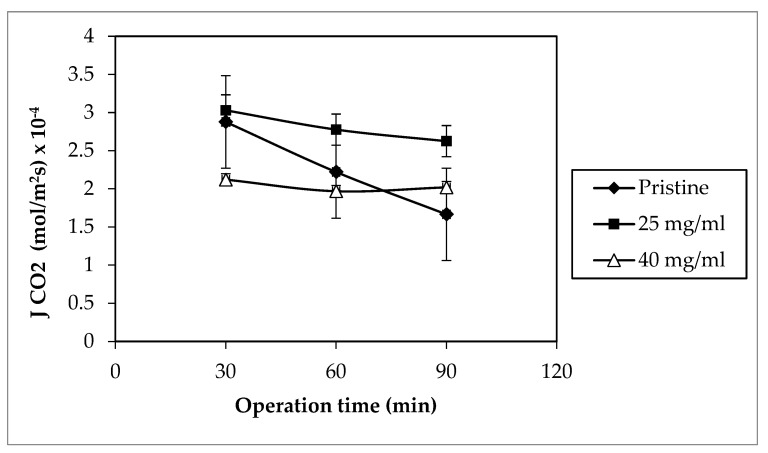
Comparison of the modified with the pristine PVDF hollow fiber membranes on a CO_2_ absorption flux (Qgas: 120 mL min^−1^; Qliquid: 100 mL min^−1^).

The CO_2_ absorption flux results for the selected membranes were averaged and are illustrated against the operation time in Figure 8 below. Based on the graph represented, 25 mg modified membrane held the highest average CO_2_ absorption flux at 3.03 × 10^−4^ mol m^−2^ s^−1^ followed by the pristine membrane with MEK at 2.88 mol m^−2^ s^−1^, while the modified membrane with MEK at 40 mg mL^−1^ held the lowest average at 2.12 × 10^−4^ mol m^−2^ s^−1^. Although the membrane hydrophobicity is crucial, the coating thickness also plays a huge role in determining the offset of the CO_2_ absorption flux, as mentioned in previous works [15,50]. In the case of 40 mg modified membrane, the membrane’s contact angle surpassed those of the other selected membranes, thus giving an edge in its hydrophobicity. High hydrophobicity enabled the membrane to withstand the wetting inflicted by the absorbent liquid. This attributed to its CO_2_ absorption flux stability, which varied slightly from 2.12 × 10^−4^ mol m^−2^ s^−1^ to 2.02 × 10^−4^ mol m^−2^ s^−1^ over the period of operation. However, the thick coating layer deteriorated the membrane hydrophobicity advantage by increasing the mass transfer resistance for gas absorption [51].

Table 5 presents the highest CO_2_ absorption achieved in the total runs of the selected membranes. As listed, the pristine hollow fiber membranes with MEK at 25 mg mL^−1^ showed a promising CO_2_ absorption flux. This could be due to its high porosity and relatively lower coating layer thickness, as portrayed in Figure 5. Although the differences in thickness were low between the membranes with MEK at 25 mg mL^−1^ and 40 mg mL^−1^, it clearly affected the CO_2_ absorption flux significantly. In addition, it could be the possible blockage of pores due to the intrusion of the coating solution. The polymer coating solution tended to get thicker, as the polymer concentration increased, allowing a higher possibility of pore blockage [35].

Despite both MEK with a concentration of 25 mg mL^−1^ and pristine membranes acquired a high flux, the membrane’s performance progressively worsened over the course of time. At 90 min, the pristine membrane CO_2_ absorption flux decreased to 1.67 mol m^−2^ s^−1^, demonstrating a 42.11% drop of flux. Owing to its low contact angle, leading to low hydrophobicity, this result was expected. Even so, 25 mg modified membrane held its high CO_2_ absorption flux with a slight drop in the initial value of 13.33% during 90 min of operation. Considering its high hydrophobicity, it resisted the degree of wetting caused by the liquid absorbent. Nevertheless, the membrane wetting occurred on both cases, affecting both MGA performances.

In addition to the results obtained, Table 6 represents the comparison of the data from this study to those of other known literatures to gauge the difference of modified membrane performances. From the flux listed in the table, the CO_2_ absorption flux in this work was distinct as compared to those in other references. The low liquid flow rate used in this work was probably one of the reasons why CO_2_ absorption fluxes differed. Other than that, a few of the references presented used 10–30 bundles of hollow fibers. This increased the surface contact area, enabling a higher CO_2_ absorption flux. Nevertheless, a single strand of PVDF hollow fiber used in this work already exceeded one of the references that comprised a higher number of fibers used. Therefore, this work could obtain higher performance with different set of parameters. Nonetheless, at a 100 mL min^−1^ liquid absorbent flow rate, the CO_2_ flux obtained in this work was proficient, even with lower parameters set.

## 4. Conclusions

The deposition of a PP coating layer on the surface of PVDF hollow fiber membrane was a success, leading to changes in its surface morphology. The changes between the modified membranes with and without the non-solvent were quite distinct, when it was viewed via SEM. The modified membranes without MEK clearly showed the inferiority in the coating due to the absence of its precipitator and binder to homogenize the PP aggregations across the membranes surface. Hence, it could lead to pore blockages, which is in an offset for an application that requires the balance in hydrophobicity and porosity. The modified membranes with MEK and a dilute PP concentration showed less presence of PP microspherulites on the surface. As the concentration increased, especifically at a PP concentration of 25 mg mL^−1^, PP microspherulites started to form. At a PP concentration of 40 mg mL^−1^, the SEM images of the modified membranes showed the highest PP aggregations, which completely covered the membrane’s surface. Spherulite aggregations depicted from the SEM images of the modified membranes were closely related with the hydrophobicity and porosity of the membranes. The highest contact angle was achieved at the highest PP concentration for both modified membranes with and without MEK, reaching up to 119.85° and 102.38°, respectively. This data proved the deposition of a PP coating layer rendered the surface hydrophobic (>90°) from its base PVDF membrane state. The highest porosity was also achieved at a 40 mg mL^−1^ PP concentration for the modified membrane with MEK. However, it could be an offset for the membrane’s performance due to possibilities of liquid intrusion into the pores in applications such as MGA and MD. Therefore, the balance between the surface hydrophobicity and the porosity was prioritized. The results for the FTIR spectra for all the modified membranes showed an indication of the PP coating presence on the surfaces with and without MEK. The only distinct characteristics were the intensity of the peaks, implying the coating homogeneity on the membrane. This led to the coating layer evaluation via MGA for the CO_2_ absorption flux. The pristine membrane and the modified PVDF hollow fiber membranes with MEK at 25 mg mL^−1^ clearly exhibited high CO_2_ fluxes, but the modified PVDF hollow fiber membranes with MEK at 40 mg mL^−1^ showed better flux stability. Nevertheless, the modified PVDF hollow fiber membranes with MEK at 25 mg mL^−1^ displayed both stability and high performance, despite a low contact angle compared to the modified PVDF hollow fiber membrane with MEK at 40 mg mL^−1^.

## Figures and Tables

**Table 1:** Specifications of the commercial poly(vinylidene fluoride) (PVDF) hollow fiber membrane.

Parameter	Specifications
Average pore size (µm)	0.03
Fiber inner diameter (mm)	0.7
Fiber outer diameter (mm)	1.3
Fiber surface area (m^2^)	1.0
Intrinsic contact angle (°)	72.53

**Table 2:** Specifications of the membrane module for the membrane gas absorption (MGA).

Parameter	Specifications
Module inner diameter (mm)	16
Module length (mm)	220
Number of fibers (n)	1
Effective fiber length (mm)	180
Effective membrane area (m^2^)	7.4 × 10^−4^

**Table 3:** Hansen solubility parameters (HSP) values for PVDF, methyl ethyl ketone (MEK), and xylene.

Sample	$\delta^{2}{}_{D,i}$ (MPa)	$\delta^{2}{}_{P,i}$ (MPa)	$\delta^{2}{}_{H,i}$ (MPa)	$\delta^{2}{}_{T,p}$ (MPa)	RED	Reference
PVDF	17.2	12.5	9.2	23.2	-	[31]
MEK	16.0	9.0	5.1	19.05	1.2	[33]
Xylene	17.4	1.0	3.1	17.70	2.64	[34]

**Table 4:** Summarized bulk porosity values and contact angles for all PP concentrations with and without MEK.

Sample	PP Concentration (mg mL^−1^)	Bulk Porosity (%)	Contact Angle (°)
Without MEK	10	78.82 ± 3.26	91.97 ± 8.20
	25	77.29 ± 1.55	100.78 ± 3.45
	40	77.25 ± 1.04	102.38 ± 3.96
With MEK	10	83.43 ± 4.61	98.79 ± 1.55
	25	80.27 ± 0.67	106.09 ± 2.02
	40	87.62 ± 1.32	119.85 ± 4.66

**Table 5:** Highest CO_2_ absorption flux over the total runs of the selected membrane samples.

Membrane Type	CO_2_ Absorption Flux (mol m^−2^ s^−1^)
Pristine	3.13 × 10^−4^
25 mg modified	3.33 × 10^−4^
40 mg modified	2.42 × 10^−4^

**Table 6:** Performance comparison of the composite membranes in this study and the literature.

Membrane Type	CO_2_ Absorption Flux (mol m^−2^ s^−1^)	Remarks	Reference
PVDF hollow fiber	2.3 × 10^−3^	Water absorbent; $Q_{l_{in}}$ = 0.9 m s^−1^ Number of fibers = 30	[24]
Polysulfone (PSf) hollow fiber	2 × 10^−4^	Water absorbent; $Q_{l_{in}}$ = 300 mL min^−1^ Number of fibers = 7	[52]
Composite PVDF	8.7 × 10^−4^	AMP absorbent; $Q_{l_{in}}$ = 100 mL min^−1^	[15]
Surface modifying macromolecule (SMM)-modified PVDF hollow fiber membrane	5.4 × 10^−3^	Water absorbent; $Q_{l_{in}}$ = 300 mL min^−1^ Number of fibers = 10	[53]
PSf + PEG200 hollow fiber membrane	1.09 × 10^−3^	Water absorbent; $Q_{l_{in}}$ = 1.4 m s^−1^ Number of fibers = 10	[54]
PP-modified PVDF hollow fiber membrane	3.33 × 10^−4^	Water absorbent; $Q_{l_{in}}$ = 100 mL min^−1^ Number of fibers = 1	This study

## Data Availability

Not applicable.
